# An in vivo comparison of wound healing characteristics of two commercial acellular dermal matrices

**DOI:** 10.1002/cre2.412

**Published:** 2021-05-03

**Authors:** Sophie R. Couto, Xianghong Luan, Jeffrey A. Rossmann, William V. Stenberg, Karen Yen, Sarah Atwi, Kathy K. Svoboda

**Affiliations:** ^1^ Department of Periodontics, Texas A&M University College of Dentistry Dallas Texas USA; ^2^ Department of Biomedical Sciences, Texas A&M University College of Dentistry Dallas Texas USA; ^3^ Texas A&M University College of Dentistry Dallas Texas USA; ^4^ Sunnybrook Health Sciences Center University of Toronto Toronto Ontario Canada

**Keywords:** biomaterial, collagen, fibroblast, periodontal surgery

## Abstract

**Objectives:**

Many acellular dermal matrices (ADMs) are available for use in periodontal surgical procedures. However, few studies exist evaluating their in vivo healing properties. The objectives of this study were to compare the wound healing and remodeling of two ADMs used for gingival augmentation procedures in the rat model.

**Materials and methods:**

This was a nonrandomized controlled split‐mouth study. Envelope flaps were surgically created in the maxillary quadrants of 24 Sprague Dawley rats. Each received either (a) AlloDerm Regenerative Tissue Matrix, or (b) OrACELL. Gingival tissue from one mandibular quadrant served as the untreated control. Six male and six female rats were treated for 7 or 21 days. Biopsies were processed for histologic analysis (H&E, Picro‐sirius red, Verhoeff's solution) or RNA analysis (RT‐PCR) to analyze the expression of type I collagen (Col1a1), fibronectin (Fn‐1) and VEGF‐A (Vegf‐A).

**Results:**

There was a greater density of fibroblasts in OrACELL compared to AlloDerm at both timepoints. There was a greater density of elastin present in AlloDerm compared to OrACELL at 7 days but no differences at 21 days. There were no differences between test groups in the percentage of birefringent collagen or in the expression of Vegf‐A or Fn‐1. At 7 days, there were significantly more fibroblasts for males in the OrACELL group compared to females. At 21 days, there was a significantly greater expression of Col1a1 for males in the OrACELL group compared to females.

**Conclusions:**

Early wound healing and remodeling of OrACELL appeared to occur more rapidly than AlloDerm and was accelerated in male rats. Whether these results have clinical implications for soft tissue grafting procedures in humans remains to be determined.

## 1 INTRODUCTION

Gingival recession affects at least one tooth in 100% of young adults, with 42% experiencing a maximum of 4 to 8 mm of recession (Seong, Newcombe, Claydon, Hellin, & West, 2018). Gingival recession can lead to dentin hypersensitivity, poor esthetics and carious or noncarious cervical lesions such as abrasions or erosions (Cortellini & Bissada, 2018). Risk factors include a thin periodontal phenotype, reduced alveolar bone thickness due to tooth malposition, and absence of attached gingiva (Zweers, Thomas, Slot, Weisgold, & van der Weijden, 2014). Other contributing factors include traumatic tooth brushing habits (Khocht, Simon, Person, & Denepitiya, 1993), trauma from lip or tongue piercings (Kapferer, Benesch, Gregoric, Ulm, & Hienz, 2007), aberrant frenum or muscle attachment (Sarfati, Bourgeois, Katsahian, Mora, & Bouchard, 2010), intrasulcular restorative margins (Kim & Neiva, 2015), orthodontic therapy (Joss‐Vassalli, Grebenstein, Topouzelis, Sculean, & Katsaros, 2010), and periodontal inflammation (Merijohn, 2016). Gingival recession also increases with age (Kassab & Cohen, 2003).

Many options exist for gingival augmentation and root coverage procedures, including autogenous tissue grafts, allografts, and xenografts. Although subepithelial connective tissue grafting is considered the gold standard for soft tissue augmentation and root coverage procedures, there are disadvantages associated with this procedure. Obtaining enough tissue in appropriate quantity and quality is one limitation. Another issue is patients generally prefer shorter, less traumatic procedures with one surgical site (Cairo, Pagliaro, & Nieri, 2008; Chambrone, Chambrone, Pustiglioni, Chambrone, & Lima, 2008; Gapski, Parks, & Wang, 2005). A second surgical site inevitably results in increased postoperative pain and discomfort with potential for neurovascular complications.

As a result of these drawbacks, use of alternative graft materials has increased. The major advantage of using an allograft or xenograft over an autograft is eliminating the need for a second surgical site. Furthermore, an unlimited quantity of tissue is available with uniform thickness allowing ideal donor tissue dimensions (Cummings, Kaldahl, & Allen, 2005).

Acellular dermal matrix (ADM) is donated human or xenogenic dermal tissue that has been rendered acellular to avoid tissue rejection. ADM includes types I, II, III and IV collagen, laminin, elastin, glycosaminoglycans and vascular channels (Cummings et al., 2005; Livesey et al., 1994). It serves as a scaffold for the ingrowth of native fibroblasts and endothelial cells to produce a de novo connective tissue matrix (Cummings et al., 2005; Wong et al., 2008). Unlike gingiva, ADM contains elastin fibers, enabling distinct visualization of the material histologically (Cummings et al., 2005). Each commercial ADM is processed using unique proprietary technology (Salvin, n.d.; Biohorizons, 2018; DentsplyImplants, 2009; Zimmer Biomet, 2018).

Of the commercial ADMs available, AlloDerm (Biohorizons, Birmingham, AL) is the most widely tested and utilized product for grafting around teeth and implants. Numerous peer‐reviewed clinical studies have compared AlloDerm to connective tissue grafting with comparable results (Aichelmann‐Reidy, Yukna, Evans, Nasr, & Mayer, 2001; Cummings et al., 2005; Gapski et al., 2005; Hirsch, Goldstein, Goultschin, Boyan, & Schwartz, 2005; Novaes Jr et al., 2001; Paolantonio et al., 2002). Only three studies, however, have compared AlloDerm to an alternative product (Barker et al., 2010; Wang et al., 2014; Wang, Suárez‐López del Amo, Layher, & Eber, 2015). Given that many commercial ADMs have been used in clinical practice for years, there is a paucity of clinical and histologic evidence comparing wound healing characteristics between products.

The number of human histologic studies using AlloDerm is limited due to ethical considerations. Cummings et al performed either a CT or AlloDerm graft at 12 teeth planned for extraction in four patients (Cummings et al., 2005). Block biopsy sections obtained after 6 months of healing revealed that both grafts were well incorporated with no gross inflammatory reaction (Cummings et al., 2005). New fibroblasts, vascular elements and collagen were present throughout the AlloDerm with retention of transplanted elastin fibers (Cummings et al., 2005). Another study demonstrated signs of revascularization, epithelial cell colonization and new collagen synthesis at 2 weeks with complete graft substitution and re‐epithelialization at 10 weeks (Scarano, Barros, Iezzi, Piattelli, & Novaes Jr, 2009). In this study, the existing collagen fibers of the AlloDerm were difficult to visualize as early as 6 weeks (Scarano et al., 2009). A recent histologic study in 22 patients who underwent breast reconstruction surgery with ADM reported colonization of the matrix with fibroblasts, myofibroblasts, lymphocytes, macrophages, multinucleated giant cells and mast cells with a relatively rapid ingrowth of blood vessels at a mean of 6 months (Bohac et al., 2018). They noted that the revascularization process of the ADM was more rapid than lymphangiogenesis; early signs of which were only evident in one patient at 9 months (Bohac et al., 2018).

A newer ADM is OrACELL (LifeNet Health, Virginia Beach, VA). In addition to maintaining its collagen and elastin, OrACELL retains native growth factors, increasing the potential for faster healing and regeneration (Salvin, n.d.). Few peer‐reviewed studies have been published. However, a recent randomized controlled trial demonstrated promising results (Vreeberg, Griffiths, & Rossmann, 2018). In this study, no significant differences in root coverage or clinical attachment level gain were observed between OrACELL and connective tissue grafting at 6 months (Vreeberg et al., 2018).

Since both AlloDerm and OrACELL have independently been reported to produce acceptable clinical outcomes for gingival augmentation procedures, we compared the two products for wound healing on a histologic and molecular level. We were interested in determining if they incorporate and remodel at similar rates and if gender has an impact on these processes. The purposes of this study were therefore to compare, in vivo, the relative gingival fibroblast density, collagen production, angiogenesis and elastin degradation associated with AlloDerm and OrACELL at two time points in males and female rats.

## 2 MATERIALS AND METHODS

### 2.1 Experimental animals

Twenty‐four Sprague Dawley rats weighing between 300 to 700 g were used for this experiment (12 males and 12 females). Rats were 6 to 9 months of age. Animal experiments were approved by the Texas A&M University College of Dentistry Institutional Animal Care and Use Committee (IACUC‐2019‐0061‐CD) in accordance with institutional guidelines. Animals were housed in pairs separated by gender with a light/dark cycle of 12 hr/12 hr.

A statistical description and power analysis (SAS System 9.4) were performed before initiating the study. The minimum number of animals required to determine a statistically significant difference between groups was determined to be 18 (*α* = 0.05, 1 − *β* = 0.8).

The primary study outcomes included gingival fibroblast distribution, collagen formation, and ADM degradation and angiogenesis at 7 and 21 days. The secondary outcome was to determine if wound healing was gender dependent.

### 2.2 Surgical procedures

#### 2.2.1 Surgical protocol

Animals were anesthetized with 3% isoflurane inhalation for 3 to 5 min followed by an intraperitoneal injection of a combination of 40–80 mg/kg Ketamine and 5–10 mg/kg Xylazine. A full‐thickness 3 mm × 3 mm pouch was created at the buccal gingival margin adjacent to the first molars in each maxillary quadrant using a spoon excavator.^1^ No vertical incisions were made (Figure S1). ADMs were prepared according to manufacturer instructions. AlloDerm was rehydrated in sterile saline in two separate baths for a total of 20–25 min until soft and pliable throughout. OrACELL was hydrated in sterile saline in a single step for 10–15 min. A 2 mm biopsy punch^2^ was taken of each prepared ADM. The surgical pouch of the maxillary right quadrant received AlloDerm and the maxillary left quadrant received OrACELL. Pouches were sealed with cyanoacrylate adhesive^3^ and hemostasis was achieved. No sutures were used.

#### 2.2.2 Postoperative care

Animals were placed under a heating lamp for post‐surgical monitoring. A subcutaneous injection of 2–5 mg/kg Nalbuphine was administered immediately post‐operatively. Criteria for early sacrifice followed IACUC recommendations, including but not limited to bleeding that could not readily be stopped, an inability to rise or move about the cage, lethargy and labored breathing. No animals were sacrificed early. Animals were observed daily for signs of distress indicating a need for humane intervention and were placed on a soft food diet^4^ for the entire postoperative period.

#### 2.2.3 Sample collection

Animals were sacrificed by carbon dioxide asphyxiation at either 7 or 21 days. Full‐thickness biopsy specimens were obtained using a 3 mm biopsy punch^5^ at the buccal gingiva adjacent to the first molars in the test and control quadrants. The biopsy punch was centered mesio‐distally at the gingiva of the first molar with the coronal aspect at the free gingival margin. A microsurgical blade^6^ was used to separate the specimen from the alveolar bone. Samples were placed in the appropriate medium depending on analysis method.

### 2.3 Histologic processing and analysis

#### 2.3.1 Histologic preparation

Biopsy specimens for histologic analysis were fixed in 10% formalin, processed and embedded in paraffin, and serially sectioned at 7 μm. Sections were stained with hematoxylin and eosin (H&E), Picro‐sirius red, or Verhoeff's solution.

#### 2.3.2 Histologic analysis

##### Fibroblast quantification

**FIGURE 1:**
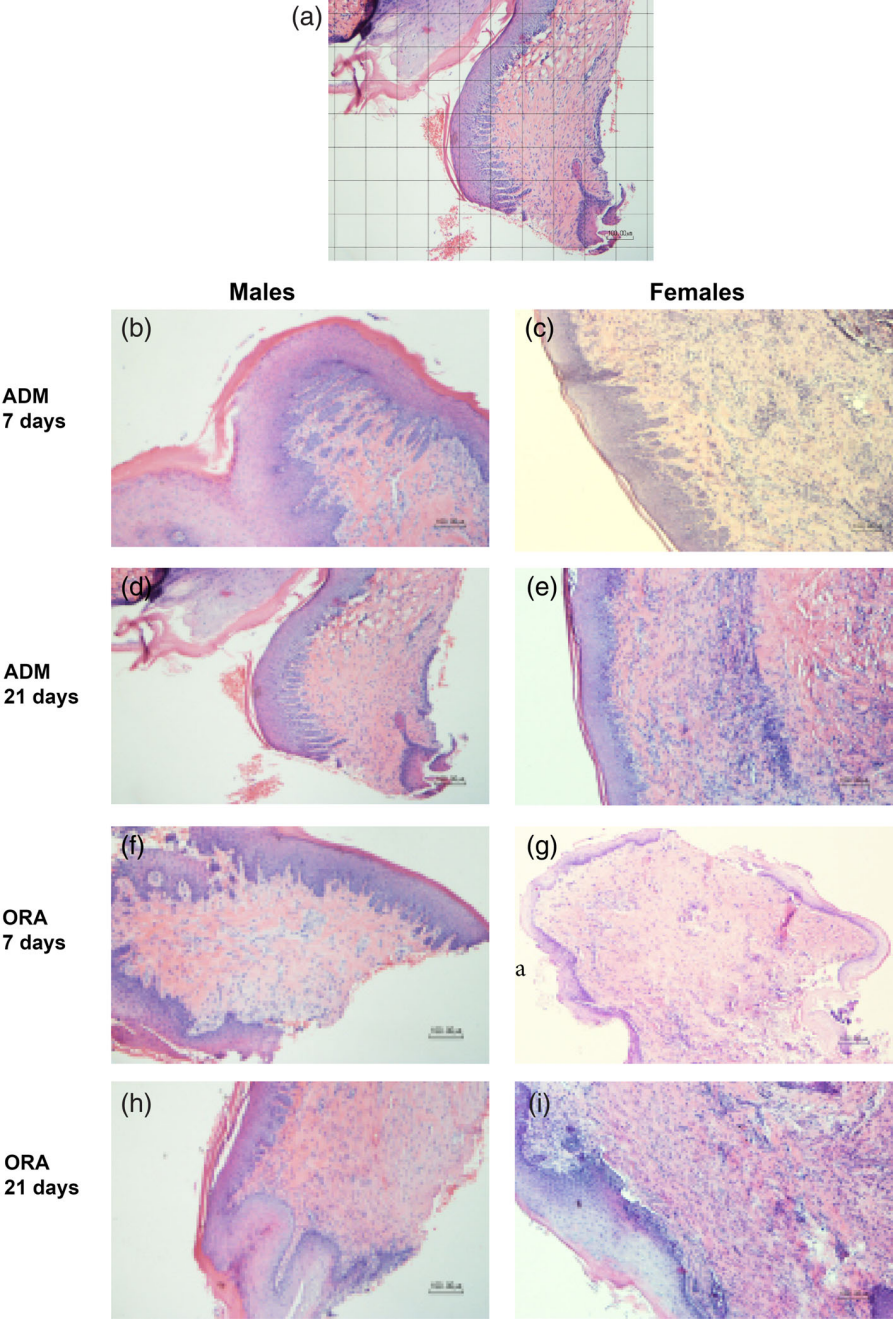
(a) Grid placement for fibroblast quantification; (b–i) Representative slides used for fibroblast quantification for AlloDerm (ADM) and OrACELL (ORA) by gender and timepoint (10× magnification, H&E stain). There was a trend towards a greater number of fibroblasts for OrACELL versus AlloDerm at 7 and 21 days. There were significantly more fibroblasts for the 7‐day males versus the 7‐day females in the OrACELL group (*p* = .041)

H&E stained slides were photographed using a light microscope with a 10× objective. The region of interest for this analysis was a defined area of the subepithelial connective tissue. Scientific imaging analysis software (ImageJ, NIH) was used to overlay a grid on each image measuring 100 μm (Cortellini & Bissada, 2018). (Figure 1a). Two adjoining boxes containing connective tissue only were selected from the center of each sample (*n* = 6/group). These were isolated from the image and divided into four squares each to facilitate counting. The fibroblasts within each box were counted by three calibrated examiners (2 were blinded to the origin of the images) (Tables 1 and 2).

**TABLE 1:** Fibroblast quantity, % birefringent collagen, and % elastin for AlloDerm, OrACELL and control groups at 7 and 21 days (mean ± *SD*)

Outcome measure	Time point	AlloDerm	OrACELL	Control	*p* value
Fibroblast (# cells)	7 days	34.03 ± 11.49	45.83 ± 19.56^c^	34.90 ± 9.96	.0495^a^
	21 days	38.28 ± 8.87	44.50 ± 7.06^c^	36.07 ± 4.84	.0494^a^
BRC (%)	7 days	44.55 ± 27.93	53.96 ± 23.37	41.42 ± 14.34	.634
	21 days	55.71 ± 19.75	52.05 ± 21.94	29.53 ± 22.50	.121
Elastin (%)	7 days	16.85 ± 6.77^b^ ^,^ ^c^	11.12 ± 3.41	6.71 ± 2.68	.0036^a^
	21 days	7.70 ± 2.63	8.09 ± 3.38	6.99 ± 2.21	.831

Abbreviation: BRC, birefringent collagen.

^a^ Indicates significant differences between groups at the indicated time point, where *p*‐value <.05.

^b^ Indicates a significant difference compared to control group.

^c^ Indicates a trend of difference between test groups from post‐hoc Tukey's HSD test where *p*‐value <.0167.

**TABLE 2:** Fibroblast quantity, % birefringent collagen, and % elastin for AlloDerm, OrACELL and control groups at 7 and 21 days by gender (mean ± *SD*)

Outcome measure	Time point (days)	AlloDerm		OrACELL		Control		*p* value
		M	F	M	F	M	F	
Fibroblast (# cells)	7	34 ± 12.07	34.05 ± 13.59	57 ± 24.07^b^	34.67 ± 1.81	37.58 ± 14.94	32.22 ± 1.78	.052^a^
	21	33.06 ± 9.18	43.5 ± 5.53	43.72 ± 9.12	45.28 ± 6.30	39.56 ± 3.56	32.58 ± 3.06	.569
BRC (%)	7	20.92 ± 19.83	60.31 ± 20.79	41.09 ± 1.63	62.54 ± 22.25	45.12 ± 4.67	38.96 ± 19.43	.131
	21	49.25 ± 17.42	62.16 ± 23.38	55.16 ± 20.01	48.93 ± 27.82	29.23 ± 14.12	29.82 ± 32.66	.820
Elastin (%)	7	7.04 ± 0.00	20.12 ± 2.15^b^ ^,^ ^c^	8.72 ± 1.40	13.52 ± 3.14^b^ ^,^ ^c^	5.03 ± 0.64	7.83 ± 3.08	.010^a^
	21	7.75 ± 3.21	7.55 ± 0.00	8.76 ± 3.29	7.08 ± 4.54	7.50 ± 1.92	6.23 ± 3.20	.484

Abbreviations: BRC, birefringent collagen; F, female; M, male.

^a^ Indicates a significant difference between genders at indicated time point.

^b^ Indicates a significant difference between genders within the same test group.

^c^ Indicates differences between genders within test groups combined, obtained from post‐hoc Welch's two sample *t* test. Significance achieved at *p*‐value <.05.

##### Collagen birefringence

**FIGURE 2:**
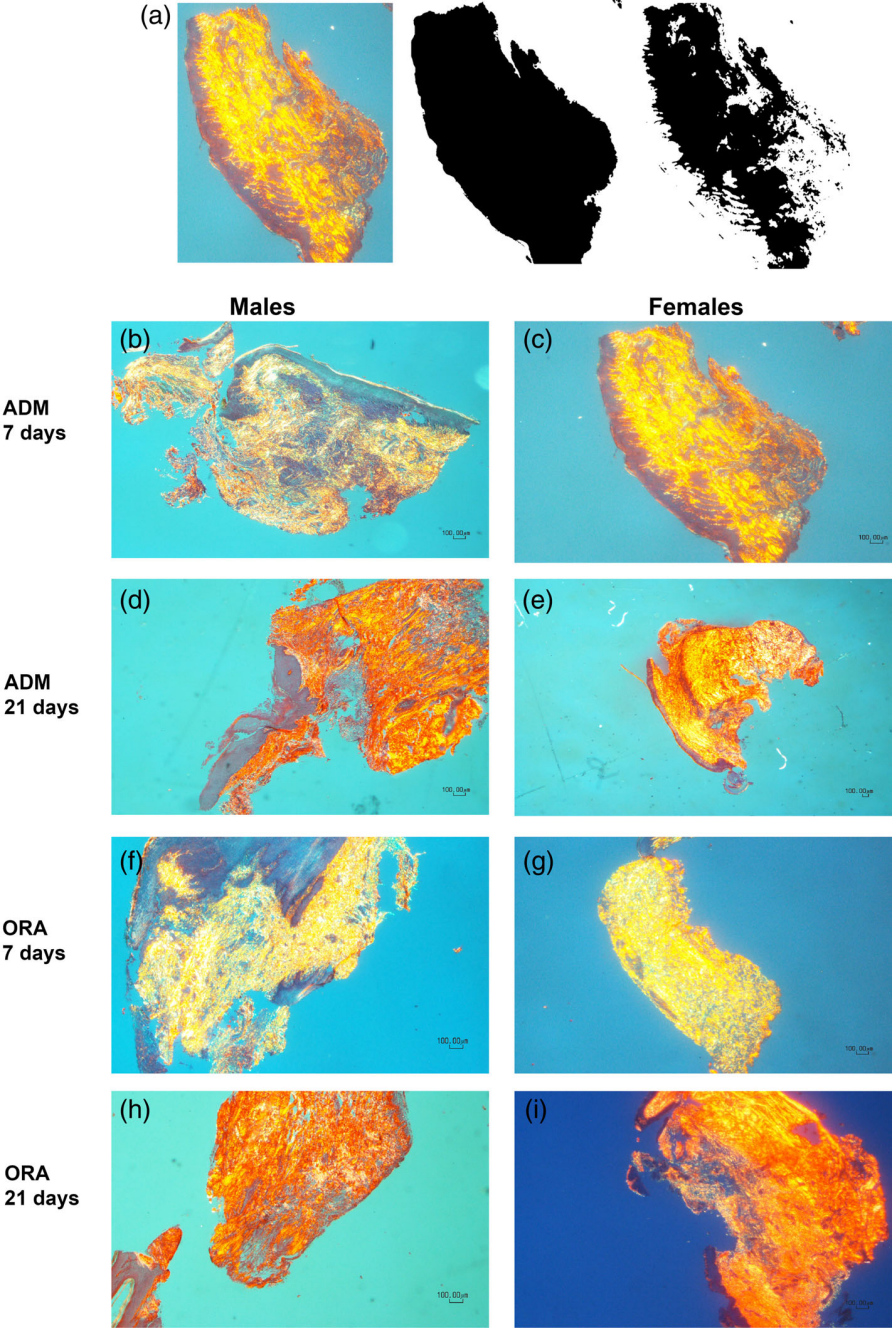
(a) Image J analysis method for % collagen birefringence calculation; (b–i) Representative slides used for % collagen birefringence analysis for AlloDerm (ADM) and OrACELL (ORA) by gender and timepoint (4× magnification, Picro‐sirius red stain). There were no statistically significant differences between ADM groups at 7 or 21 days. There were no statistically significant differences between males versus females

Picro‐sirius red stained slides were photographed using a polarizing light microscope with a 4× objective. The region of interest for this analysis was the total subepithelial connective tissue area. Scientific imaging analysis software (ImageJ, NIH) was used to create binary images to quantify the total number of black pixels representing the total tissue area (Figure 2a). A second image was then produced by separating the original image into its red, blue and green channels, and converting the red channel to binary for quantification of the Picro‐sirius red‐stained collagen. This produced a ratio of birefringent collagen to the total sample, enabling calculation of the % of collagen present in each sample (Tables 1 and 2).

##### Elastin degradation

**FIGURE 3:**
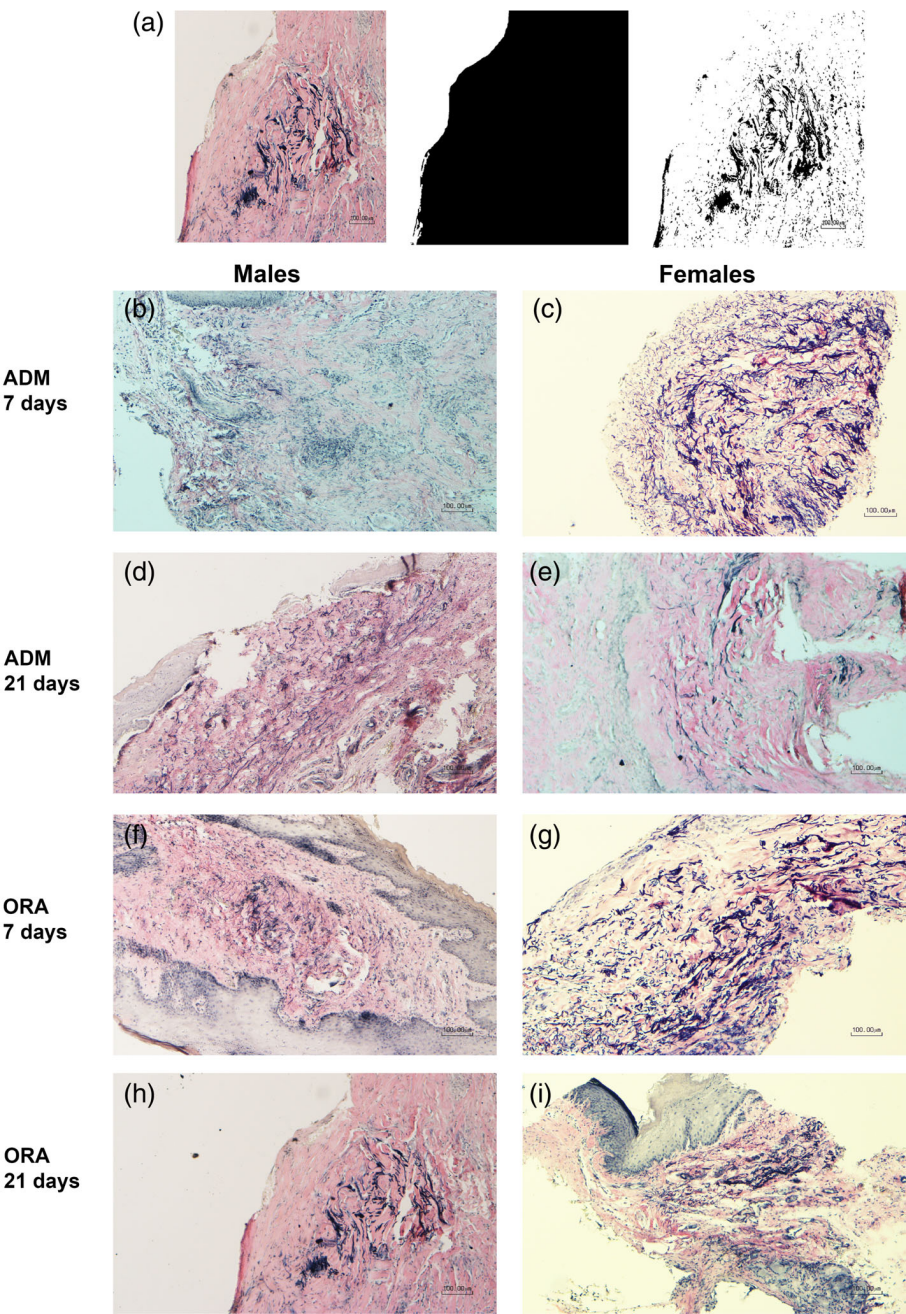
(a) Image J analysis method for % elastin quantification; (b–i) Representative slides used for % elastin for AlloDerm (ADM) and OrACELL (ORA) by gender and timepoint (10× magnification, Verhoeff's solution). There was a trend towards a greater % of elastin for AlloDerm versus OrACELL at 7 days. There was a significantly greater % of elastin for ADM groups of the 7‐day females vs. 7‐day males (*p* = .006)

Verhoeff's stained slides were photographed using a light microscope with a 10× objective. The region of interest for this analysis was the total subepithelial connective tissue area. Scientific imaging analysis software (ImageJ, NIH) was used to create binary images to quantify the total number of black pixels representing the total area of connective tissue (Figure 3a). A second image was then produced by separating the original image into its red, blue and green channels, and converting the blue channel to binary for quantification of the Verhoeff‐stained elastin. This produced a ratio of elastin and cell nucleus elements to the total connective tissue, enabling calculation of the % of elastin present in each sample (Tables 1 and 2).

### 2.4 Real‐time polymerase chain reaction (RT‐PCR)

The relative expression of genes for alpha‐1 type 1 collagen (Col1a1), vascular endothelial growth factor (Vegf‐A), and fibronectin (Fn‐1) was assessed by RT‐PCR. Biopsy specimens were stored at −80°C. Total RNA was isolated^7^ then converted to cDNA.^8^ Real‐time PCR was performed using sequence specific primers. Samples were normalized to levels of GAPDH or β‐Actin. To quantify relative differences in mRNA expression, the comparative CT method (ΔΔCT) was used to determine relative quantity. Values were graphed as the mean expression level ± *SD*. Primers used: Rat Col1a1 Forward 5′‐aatggtgctcctggtattgc‐3′, Reverse 5′‐ggttcaccactgttgccttt‐3′; Fn Forward 5′‐catgaagggggtcagtccta‐3′, Reverse 5′‐gtccattccccttttccatt‐3′; Vegf‐A Forward 5′‐cgaacagagagagggacagg‐3′, Reverse 5′‐cgactggtccgatgaaagat‐3′; β‐actin Forward 5′‐agccatgtacgtagccatcc‐3′, Reverse 5′‐accctcatagatgggcacag‐3′; GAPDH Forward 5′‐aagggctcatgaccacagtc‐3′, Reverse 5′‐ggatgcagggatgatgttct‐3′.

### 2.5 Statistical analyses

Linear mixed effects models were used to quantitate differences in average fibroblast count, % birefringent collagen, % elastin, Col1a1 mRNA, Vegf‐A mRNA, and Fn‐1 mRNA between three groups: AlloDerm, OrACELL, and control at 7 and 21 days. Significance was set at *p*‐value <.05. For linear mixed effects models that met significance, post‐hoc Tukey's HSD tests were performed to determine which group(s) had differences; significance was set at *p*‐value <.0167 (0.05 ÷ 3) to correct for multiple comparisons (Bonferroni correction). Further, we used linear mixed effects models to consider differences between genders at 7 and 21 days for each group. Significance was set at *p*‐value <.05. Significant models were followed by post‐hoc Welch's two sample *t* tests to determine average differences between genders for each group; significance was set at *p*‐value <.05.

## 3 RESULTS

### 3.1 Histology

#### 3.1.1 Fibroblast quantification

The H&E stained sections contained the epithelium and the underlying lamina propria (Figure 1). There were some staining differences between biopsied samples, but the fibroblasts in the ADM areas were identifiable. Using the randomized square approach described in the methods, three individuals counted the fibroblasts. There was a significant difference in the fibroblasts present between the AlloDerm, OrACELL, and control groups at 7 (*p* = .0495) and 21 days (*p* = .0494). Following correction for multiple comparisons, there were no significant differences between groups at either time point (*p* > .0167); however, a trend towards more fibroblasts in OrACELL than AlloDerm at both 7 days (*p* = .072) and 21 days (*p* = .058) was recorded (Table 1).

There was also a difference in fibroblasts between 7‐day male and female rats (*p* = .052). Post‐hoc analysis revealed significantly more fibroblasts in males with OrACELL than females (*p* = .041). There were no significant differences in fibroblast numbers between the 21‐day male and female animals (*p* = .569) (Table 2).

These results show differences between ADMs and gender at both early (1 week) and later (3 weeks) healing times. The increased fibroblasts in this type of tissue may lead to faster elastin degradation and collagen deposition. Based on these results we analyzed the collagen in the surgery sites.

#### 3.1.2 Collagen birefringence

To analyze the total collagen in the biopsies, we used Picro‐sirius red with polarizing light to determine the sample properties (Figure 2). Binary images were used to quantify the amount of collagen in the total tissue area. The ratio of birefringent collagen was compared to the total sample, which produced a calculation of the percent of collagen present (*n* = 3/sample) (Figure 2a). We found no differences in % collagen between the AlloDerm, OrACELL, and control groups at 7 days (*p* = .634) or between the three groups at 21 days (*p* = .121) (Table 1).

There were no differences in % collagen by birefringence between the 7‐day male and female animals (*p*= .131) or between the 21‐day male and female animals (*p* = .820) (Table 2).

This approach measures all collagen in the samples and does not discriminate between the 29 collagen types. However, type I collagen was the most abundant component. The data supports the hypothesis that the gingiva responds to the ADMs by reorganizing the lamina propria making the extracellular matrix more like the natural gingival lamina propria as there was no significant difference between the ADM groups and the control. In addition, there were no differences between males and females.

#### 3.1.3 Elastin degradation

Elastin is present in dermal tissues but not in gingival tissues (Cummings et al., 2005). In the ADM grafts, the existing elastin was degraded over time (Richardson, 2002). Therefore, staining for elastin was used to determine how active the cells were degrading it. We found a significant difference in the % of elastin between the AlloDerm, OrACELL, and control groups at 7 days (*p* = .0036). Post‐hoc analysis revealed that the % of elastin present was significantly greater for the AlloDerm compared to the controls (*p* = .003). There was also a trend for greater elastin present for the AlloDerm compared to the OrACELL (*p* = .074). However, there was no difference in the % of elastin between groups at 21 days (*p* = .831) (Table 1).

There was a significant difference for the % of elastin between the 7‐day male and female animals (*p* = .010). Following post‐hoc analysis, the gender difference was significant for the combined test groups (AlloDerm + OrACELL). There was significantly greater elastin present in 7‐day females compared to 7‐day males (*p* = .006). However, there was no difference in elastin between the 21‐day male and female animals (*p* = .484) (Table 2).

These results support the conclusion that the ADMs have more elastin than normal gingival tissue and that AlloDerm has more than OrACELL. This data also shows that by 21 days, the elastin had decreased in both ADMs. The difference between males and females support the observation that the males had more elastin degradation in 1 week, but by 3 weeks both groups were similar.

### 3.2 RT‐PCR

#### 3.2.1 Alpha‐1 type 1 collagen (Col1a1)

**FIGURE 4:**
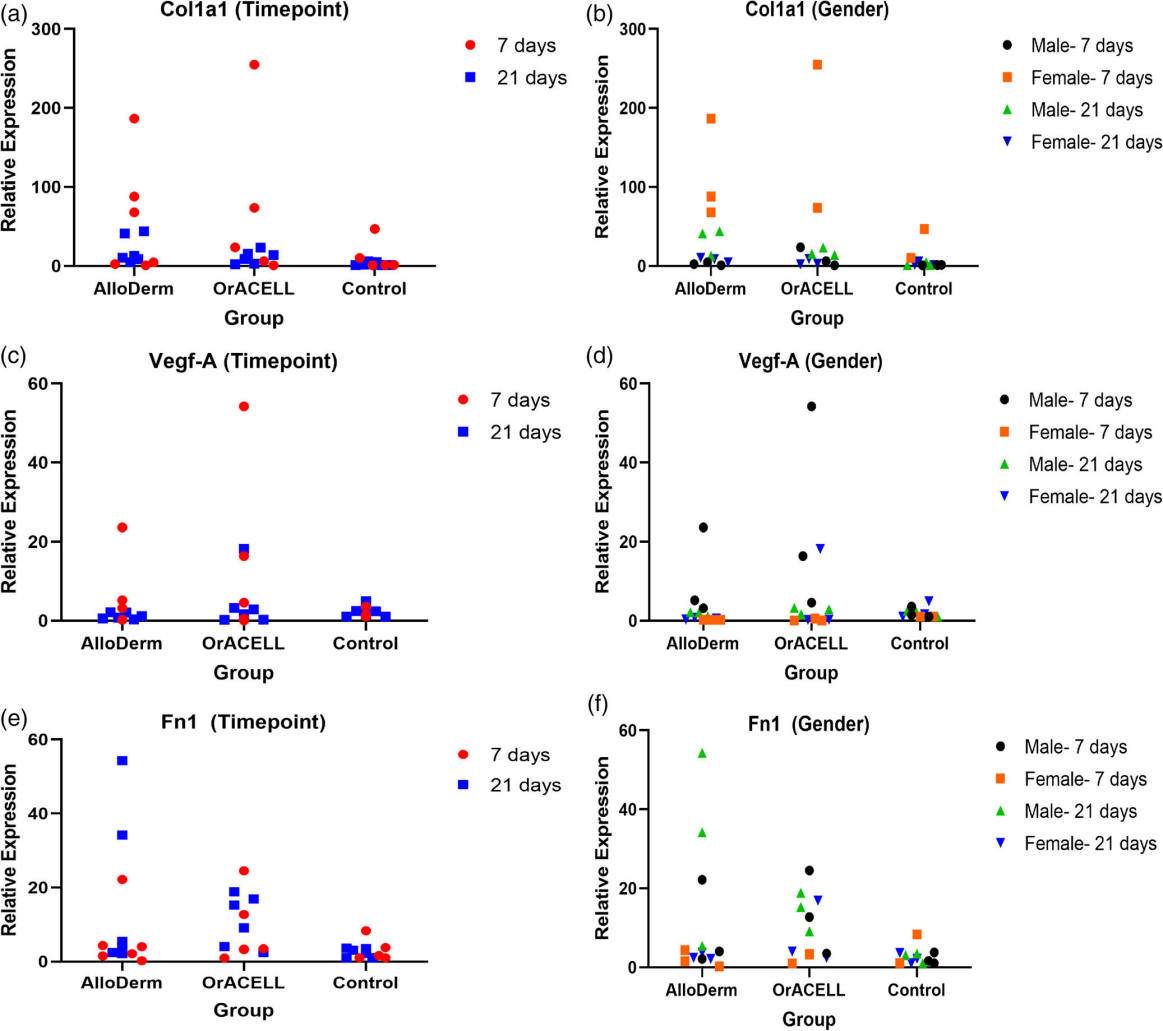
(a–f) RT‐PCR results for relative Col1a1 (a, b), Vegf‐a (c, d), and Fn‐1 (e, f) expression. Trend of greater Col1a1 expression for test groups of 7‐day females versus 7‐day males. Significantly greater Col1a1 expression for 21‐day males versus 21‐day females in the OrACELL group (*p* = .027). Trend of greater Vegf‐A expression for test groups of 7‐day males versus 7‐day females. Trend of greater Fn‐1 expression for test groups of 21‐day males versus 21‐day females

We used RT‐PCR to quantitate type I collagen in biopsies from half of the surgical sites. We found no differences in expression of Col1a1 between the AlloDerm, OrACELL, and control groups at 7 days (*p* = .176), but there was a significant difference between groups at 21 days (*p* = .017). Post‐hoc analysis demonstrated increased Col1a1 expression in the AlloDerm group compared to controls at 21 days (*p* = 0.013) (Figure 4a).

**FIGURE 5:**
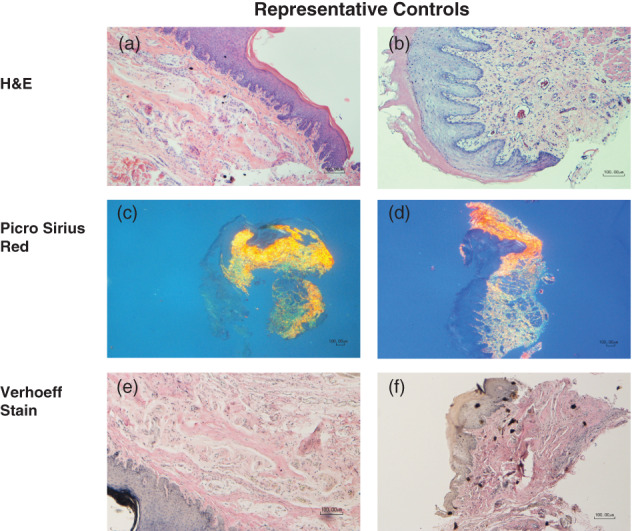
(a–f) Representative images from the control group. (a) 7‐day male (H&E, 10×); (b) 21‐day female (H&E, 10×); (c) 7‐day female (Picro Sirius red, 4×); (d) 21‐day female (Picro Sirius red, 4×); (e) 7‐day male (Verhoeff's solution, 10×); (f) 21‐day male (Verhoeff's solution, 10×)

There was a significant difference in the expression of Col1a1 between the 7‐day male and female animals (*p* = 0.028). When post‐hoc analysis was performed, this difference was not significant though there was a trend when the two test groups were combined (AlloDerm + OrACELL); there was greater expression of Col1a1 for the ADM groups in the 7‐day female subjects compared to the test groups in the 7‐day males (*p* = .060). There was a significant difference in the expression of Col1a1 between the 21‐day male and female animals (*p* = .013). Following post‐hoc testing, the difference was in the OrACELL group, with increased Col1a1 in 21‐day males compared to 21‐day females (*p* = .027) (Figure 4b).

Interestingly, even though the Picro‐sirius red analysis did not show a difference between samples, the mRNA data demonstrated that ADM fibroblasts produced type I collagen. In addition, early wound healing (7 days) responded the same and were comparable to controls. However, by 21 days there was a difference between ADM groups and gender also affected the collagen mRNA levels.

#### 3.2.2 Vascular endothelial growth factor A (Vegf‐A)

Measuring the differences in angiogenesis could be achieved several ways, such as staining for endothelial cells, or counting vessels in H&E stained sections. We chose to measure the amount of Vegf‐A mRNA in the samples. We found no differences in the expression of Vegf‐A between the AlloDerm, OrACELL, and control groups at 7 days (*p* = .3496) or between the three groups at 21 days (*p* = .415) (Figure 4c).

However, there was a trend towards a significant difference in the expression of Vegf‐A between the 7‐day male and female animals (*p* = .0529). Following post‐hoc testing, there was a trend when comparing the two test groups combined (AlloDerm + OrACELL); there was greater expression of Vegf‐A for the test groups in the 7‐day male subjects compared to the test groups in the 7‐day females (*p* = .078). There were no differences in the expression of Vegf‐A between the 21‐day male and female animals (*p* = .620) (Figure 4d).

These results indicate that both ADMs attracted endothelial cells and supported the formation of new blood vessels. In addition, by 7 days the tissues were expressing the same amount of Vegf‐A as control tissue.

#### 3.2.3 Fibronectin 1 (Fn‐1)

Fibronectin, a provisional extracellular matrix protein produced by fibroblasts was used as a third marker in the study. We found no differences in the expression of Fn‐1 between the AlloDerm, OrACELL, and controls at 7 (*p* = .529) or 21 days (*p* = .1452) (Figure 4e).

In addition, there was no difference in the expression of Fn‐1 between the 7‐day male and female animals (*p* = 0.110) or the 21‐day male and female animals (*p* = .058). Following post‐hoc analysis, a trend was noted when comparing the test groups combined (AlloDerm + OrACELL), with greater expression of Fn‐1 for the ADM test groups in the 21‐day males compared to the 21‐day females (*p* = .072) (Figure 4f).

These results indicate that both ADMs had a similar provisional extracellular matrix profile and it was comparable to controls. In addition, gender did not affect the expression of fibronectin in the ADM test tissues.

## 4 DISCUSSION

The inspiration for this research was the in vitro study by Richert et al which found no significant differences in rat gingival fibroblast growth and distribution between AlloDerm, Puros Dermis, and PerioDerm (Richert, 2016). We decided to compare OrACELL, an ADM with sparse supporting literature to AlloDerm, an ADM with abundant supporting literature and hypothesized that there would be no differences in their wound healing properties. However, this in vivo study found several differences between the two ADMs, with a trend for a higher number of fibroblasts at 7 and 21 days and less elastin at 7 days in the OrACELL group. OrACELL appeared to have faster early wound healing characteristics than AlloDerm. No signs of inflammation were noted at either time point.

The rat model was chosen for several reasons. Histologic studies in humans present with numerous constraints. Firstly, surgery must be performed at sites planned for extraction, leading to sample size limitations. Additionally, there could be challenges regarding compliance with post‐operative instructions and follow‐up appointment attendance. Rats are a well‐established animal model with gingival anatomy suitable for experimental investigation. However, it has been shown that wound healing in rats was more rapid than humans (Weber et al., 2019). The clotting time in rats is three times faster, leading to faster wound stability (Weber et al., 2019). Rats could also synthesize their own Vitamin C, a necessary cofactor for collagen synthesis (Weber et al., 2019).

Most experiments only use male animals, resulting in less translational research applicable to both men and women. Male and female rats were included in this study to evaluate gender differences. In rats as well as humans there are gender distinctions in important components of wound healing (Weber et al., 2019). For example, gender differences exist in relation to the coagulation system, specifically prothrombin time (PT), activated partial thromboplastin time (aPTT), thrombin time (TT) and fibrin values (Weber et al., 2019). Adult female rats are also significantly smaller than males, resulting in fewer cells available for matrix colonization and remodeling (Harkness, VandeWoude, & Wheler, 2013). This study found that gender did have an impact on wound healing characteristics, with faster early wound healing in male compared to female rats. This may have translational applications to the clinician, for example, female patients may benefit from adjunctive use of biologics or growth factors, or simply from leaving sutures in for longer periods of time.

Timepoints of 7 and 21 days were chosen because existing in vitro studies examining ADMs observed gingival fibroblast migration at these times (Maia et al., 2011; Richert, 2016; Rodrigues et al., 2010). For example, Rodrigues et al seeded AlloDerm with human gingival fibroblasts and evaluated cell distribution at 7, 14 and 21 days (Rodrigues et al., 2010). Although there was limited migration into the matrix, cell adhesion and spreading were evident as early as 7 days (Rodrigues et al., 2010). Existing in vivo histologic studies on AlloDerm had greater variation in study duration, examining surgical sites from 3 days to 9 months (Bohac et al., 2018; Cummings et al., 2005). Human studies have demonstrated signs of revascularization, epithelial cell colonization and new collagen synthesis at 2 weeks (Scarano et al., 2009).

In this study, at 7 and 21 days, there were more fibroblasts in the OrACELL compared to AlloDerm. Since both materials were acellular, all cells present histologically were derived from host gingival tissue. More fibroblasts, the primary cells responsible for ECM production, signified rapid migration and active proliferation phase for faster wound healing. Increased fibroblasts may lead to fibrosis or scaring in dermal tissues, but in oral tissues these pathologies usually do not occur (Johnson, Francis, & DiPietro, 2014; Stephens, Davies, al‐Khateeb, Shepherd, & Thomas, 1996). LifeNet Health, the manufacturer of OrACELL, advertised that the product retains its native growth factors (Salvin, n.d.). Though they do not specify which growth factors were present, multiple growth factors attract fibroblasts, including fibroblast growth factor (FGF), platelet‐derived growth factor (PDGF), and transforming growth factor β (TGF‐β) (Alavi, 2019). This property is clinically and economically advantageous, as products containing growth factors are often used adjunctively in periodontal plastic surgery to improve healing.

Although there were no significant differences between ADM test groups in the percentage of birefringent collagen or in the expression of Col1a1, there was a trend towards more Col1a1 expression in the OrACELL group at 7 days. The biopsy material for RNA extraction included both epithelium and underlying stroma. The presence of the epithelium may have diluted the mRNA, but all samples were treated the same. In future experiments, separating the epithelium from the stroma may provide more precise results but tissue processing may also cause RNA degradation. If the ADMs were left in place longer or more samples were compared the results may be different.

In addition to the potential growth factors associated with OrACELL, inherent structural differences of the two products may have contributed to these results. For example, the OrACELL was thicker than the AlloDerm. A range of thickness exists for both matrices, with AlloDerm ranging from 0.9 to 1.6 mm and OrACELL ranging from 0.76 to 1.75 mm thick (Salvin, n.d.; Biohorizons, 2016). However, greater tissue thickness means a greater distance for native cells to migrate. The connective tissue scaffold of OrACELL may have been denser, with an enhanced framework for cell migration; or more rigid, with better space maintenance than AlloDerm. A future ultrastructural or biomechanical study of both products would aid in elucidating these characteristics.

At 7 days there was trend towards greater elastin density in the AlloDerm compared to the OrACELL group. Unlike dermal tissue, gingival tissue does not contain elastin (Cummings et al., 2005). The presence of elastin meant that (a) the ADM had been successfully implanted and remained in place, and (b) we could assess elastin remodeling. Unlike the attached gingiva, the nonkeratinized oral mucosa does contain a small amount of elastin, explaining the elastin staining in the control group (Figure 5). (Hsieh, Chang, Huang, Liao, & Yuan, 2010).

It is possible that we observed more elastin in AlloDerm because the original product contained more. Alternatively, OrACELL may have faster early elastin remodeling, supported by more fibroblasts. By 21 days, there were no significant differences between groups, indicating that both products reach the same stage of remodeling in 3 weeks. In a human study, Cummings et al noted retained elastin fibers after 6 months (Cummings et al., 2005). In contrast, Richardson and Maynard demonstrated a significant reduction in the dimension of elastic fibers after 4 months in humans (Richardson, 2002). Therefore, it is still unclear if elastin from implanted ADMs becomes completely remodeled and whether this has clinical significance for soft tissue procedures using allografts. The tendency of the MGJ to rebound to its original position after a free gingival graft is thought to induce a coronal displacement of the soft tissue margin, or creeping attachment (Agudio, Chambrone, & Pini Prato, 2017). The elastin fibers of the oral mucosa may play a role in this phenomenon.

An additional objective of this study was to assess differences between groups in angiogenesis and revascularization. Therefore, we measured the gene expression levels of VEGF‐A. VEGF, a cytokine produced by platelets, endothelial cells, neutrophils and macrophages stimulates the migration and proliferation of endothelial cells for angiogenesis (Alavi, 2019; Schwartz et al., 2015). Since OrACELL was prepared to retain native growth factors, we expected a higher expression of Vegf‐A in the OrACELL group. At both time points there was slightly greater Vegf‐A expression for OrACELL compared to AlloDerm, but the levels were not significantly different.

Fibronectin, an ECM protein produced by fibroblasts important in wound healing, was not significantly different between groups (Alavi, 2019). It stabilized the initial clot, guided cell migration to the site of injury, and was a large component of the early extracellular provisional wound matrix (Alavi, 2019; Schwartz et al., 2015). Fibronectin gene expression was expected to increase through the maturation phase. In this study both AlloDerm and OrACELL displayed elevated Fn‐1 expression relative to the control, with greater expression at 21 days compared to 7 days.

A secondary objective of this study was to determine if wound healing was gender dependent. There were no significant gender differences between either ADM product in the percentages of birefringent collagen or elastin, or expression of Vegf‐A and Fn‐1. Since no differences were observed for elastin, we concluded that elastin remodeling was similar in males and females. There was a trend towards a greater Col1a1 expression for females in both ADM groups at 7 days but not at 21 days. A possible explanation for this could be hormonal influences as we did not track the estrous cycles of the females. Additionally, if the epithelial layer was thicker for the male group the results may have been diluted.

In the OrACELL group, there were significantly more fibroblasts in males at 7 days, and a significantly greater expression of Col1a1 in males at 21 days. A similar trend was observed for Fn‐1 expression in both ADM groups. Males had significantly more fibroblasts producing more collagen and fibronectin. Although a standardized diameter tissue punch was used for all biopsies, the specimen thicknesses were not uniform. Larger rats would be expected to have thicker tissue with more numerous cellular components, producing greater expression of Col1a1. In this study the average weight of the male rats was approximately twice that of the female rats, an important disparity which may explain these differences.

This study had several limitations. The first was that the sample size was small. The second was that the control group was untreated. Creating a surgical pouch without implanted tissue would produce tissue injury to more accurately assess wound healing differences. In the study by Cummings et al, a coronally advanced flap was used as the control group (Cummings et al., 2005). A primarily qualitative analysis was performed, making accurate comparisons to the present study difficult. A third limitation was that test and control quadrants were not randomized; test quadrants were always located in the maxilla and control quadrants in the mandible. Because the buccal gingiva of the mandible was thinner and more difficult to access, test groups were standardized to the maxilla.

No attempt was made for acellular dermal matrix orientation, despite both companies recommending a specific orientation of the dermis. For example, the manufacturer's instructions for OrACELL state that the reticular side should be placed against the surgical wound or most vascularized tissue with the papillary side facing up (Salvin, n.d.). Though only tested using AlloDerm, it was demonstrated in human clinical studies that the orientation of the dermis had no effect on the amount of root coverage achieved (Henderson et al., 2001). Whether this produces histologic differences remains unknown.

This in vivo study found several differences between AlloDerm and OrACELL, two commercially available acellular dermal matrices used for periodontal soft tissue grafting procedures. OrACELL had more fibroblasts at 7 and 21 days and less elastin at 7 days. OrACELL had faster early wound healing characteristics compared to AlloDerm within the limits of this study.

## 5 CONCLUSION

This split‐mouth histologic and molecular study found differences in the wound healing characteristics of two ADMs available for use in periodontal surgery; AlloDerm and OrACELL. OrACELL exhibited faster fibroblast migration to the injury site as well as faster elastin remodeling. A potential explanation for this may be the presence of retained growth factors. A gender difference was also observed in the response to these implanted materials. Males displayed faster elastin remodeling and wound stabilization, and greater angiogenesis than females. This reinforces the current understanding that gender distinctions exist in wound healing (Weber et al., 2019).

Whether these results have positive or negative clinical implications for soft tissue grafting procedures in humans remains to be determined. AlloDerm is a time‐tested product supported by a large body of evidence. Future studies should focus on comparing OrACELL to its predecessor to determine if it can produce equivalent or superior long‐term clinical results.

## CONFLICT OF INTEREST

The authors report no conflicts of interest.

## AUTHOR CONTRIBUTIONS

Sophie R. Couto, Xianghong Luan, Jeffrey A. Rossmann, William V. Stenberg and Kathy K. Svoboda (mentor) designed the experiment. Sophie R. Couto performed the animal surgeries, analyzed the data and wrote the manuscript. Xianghong Luan and Karen Yen performed and analyzed the RT‐PCR data. Sarah Atwi performed the statistical analysis. All authors edited the manuscript.

## Supporting information


**Figure S1.** Envelope flap preparation prior to placement of ADM material A and B, (arrow); C, AlloDerm and D, OrACELL prior to hydration and implantation.

## Data Availability

The data that support the findings of this study are available from the corresponding author upon reasonable request.

## ENDNOTES

^1^
Thompson Dental, 1.15 mm.

^2^
Premier Uni‐Punch.

^3^
PeriAcryl, GluStitch Inc.

^4^
DietGel, ClearH_2_O.

^5^
Premier Uni‐Punch.

^6^
Salvin Dental Specialties, Mini Blade #69.

^7^
RNeasy Plus Mini Kit, Qiagen.

^8^
EcoDry Premix, Takara Bio Inc.
