# Supplementary material for: An in vivo comparison of wound healing characteristics of two commercial acellular dermal matrices
Source: Clin Exp Dent Res. 2021 May 3;7(5):679–91. doi: 10.1002/cre2.412 (PMC8543485; doi:10.1002/cre2.412)
Supplement: Supplementary file 1 — Figure S1. Envelope flap preparation prior to placement of ADM material A and B, (arrow); C, AlloDerm and D, OrACELL prior to hydration and implantation. [file CRE2-7-679-s001.pdf]

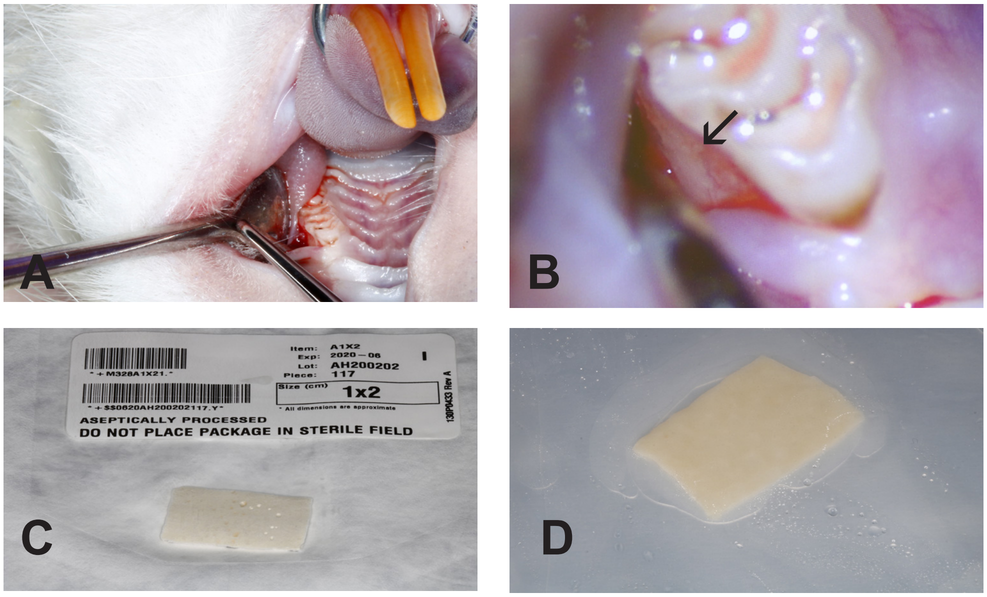

FIGURE S1: Envelope flap preparation prior to placement of ADM material (A-B, arrow); (C) AlloDermTM and (D) OrACELLTM prior to hydration and implantation.
